# Supplementary material for: Differential prevalence and prognostic value of metabolic syndrome components among patients with MASLD
Source: JHEP Rep. 2024 Aug 22;6(12):101193. doi: 10.1016/j.jhepr.2024.101193 (PMC11617726; doi:10.1016/j.jhepr.2024.101193)
Supplement: Supplementary file 1 — Multimedia component 1 [file mmc1.pdf]

Differential prevalence and prognostic value of metabolic syndrome components among patients with MASLD

Jesse Pustjens, Laurens A. van Kleef, Harry L.A. Janssen, Robert J. de Knegt, Willem P. Brouwer

Table of content

Table S1……………………………………………………………………………………2

Table S1: NHANES 2017-2020 participants characteristics among the MASLD population

	N = 2618
**Demographics**	
Age (years), median [QR]	52.9 [40.9 – 64.9]
Male, N (%)	1,424 (54.4)
Race	
- White	954 (36.4)
- Black	534 (20.4)
- Hispanic	714 (27.3)
- Asian	284 (10.8)
- Other	132 (5.0)
Years of education, median	
- <9th grade	210 (8.2)
- 9th-11th grade	260 (10.1)
- High school	636 (24.8)
- College graduate	1,458 (55.9)
Currently smoking, N (%)	361 (13.8)
Alcohol use (g/day), mean (SD)	2.89 (5.6)
**MASLD criteria, mean (SD)**	3.28 (1.2)
Obesity [a]	2565 (98.0)
(pre)Diabetes [b]	1730 (66.1)
Hypertension, N (%) [c]	1664 (63.6)
Hypertriglyceridemia , N (%) [d]	1565 (59.8)
Low-HDL, N (%) [e]	1062 (40.6)
**Biometrics**	
BMI (Kg/M^2), median [IQR]	
- Males	31.6 [28.3 – 25.6]
- Females	33.4 [29.0 – 39.5]
Waist circumference (cm), median [IQR]	
- Males	
- Females	109.6 [101.0 – 119.9]
**Biochemistry**	
ALT (U/L), median [IQR]	21 [15 – 31]
AST (U/L), median [IQR]	20 [16 – 25]
HbA1c (%), median [IQR]	6 [6-6]
Platelets	243 [205 – 285]
**FibroScan®**	
Liver Stiffness Measurements (kPa)	5.6 [4.5 – 7.0]
Controlled Attenuation Parameter (dB/m)	316 [294 – 347]

[a] BMI ≥ 25 kg/m^2 or WC > 94 cm Waist circumference >94 cm (males), > 80 cm (females), or ethnically adjusted
[b] Fasting serum glucose ≥ 5.6 mmol/L or 2-hour post-load glucose levels ≥ 7.8 mmol/L or HbA1c ≥ 5.7% OR type 2 diabetes or treatment for type 2 diabetes
[c] Blood pressure ≥ 130/85 mmHg or specific antihypertensive drug treatment
[d] Plasma triglycerides ≥ 1.70 mmol/L or lipid lowering treatment
[e] Plasma HDL-cholesterol ≤ 1.0 mmol/L (males) and ≤ 1.3 mmol/L (females) OR lipid lowering treatment

Note: Data are presented as mean (SD), median [P25-P75], or n and percentage.
Abbreviations: NHANES, National Health and Nutrition Examination Survey; MASLD, metabolic-dysfunction steatotic liver disease; ALT, alanine aminotransferase; AST, aspartate aminotransferase; P25-P75, 25th–75th percentile;
