# Supplementary material for: Differential prevalence and prognostic value of metabolic syndrome components among patients with MASLD
Source: JHEP Rep. 2024 Aug 22;6(12):101193. doi: 10.1016/j.jhepr.2024.101193 (PMC11617726; doi:10.1016/j.jhepr.2024.101193)
Supplement: Multimedia component 2 [file mmc2.docx]

**Journal of Hepatology**

**CTAT methods**

Tables for a “Complete, Transparent, Accurate and Timely account” (CTAT) are now mandatory for all revised submissions. The aim is to enhance the reproducibility of methods.

- Only include the parts relevant to your study
- Refer to the CTAT in the main text as ‘Supplementary CTAT Table’
- Do not add subheadings
- Add as many rows as needed to include all information
- Only include one item per row

**If the CTAT form is not relevant to your study, please outline the reasons why:**

|  |
| --- |

- 1. **Antibodies**

| **Name** | **Citation** | **Supplier** | **Cat no.** | **Clone no.** |
| --- | --- | --- | --- | --- |
|  |  |  |  |  |

- 1. **Cell lines**

| **Name** | **Citation** | **Supplier** | **Cat no.** | **Passage no.** | **Authentication test method** |
| --- | --- | --- | --- | --- | --- |
|  |  |  |  |  |  |

- 1. **Organisms**

| **Name** | **Citation** | **Supplier** | **Strain** | **Sex** | **Age** | **Overall n number** |
| --- | --- | --- | --- | --- | --- | --- |
|  |  |  |  |  |  |  |

- 1. **Sequence based reagents**

| **Name** | **Sequence** | **Supplier** |
| --- | --- | --- |
|  |  |  |

- 1. **Biological samples**

| **Description** | **Source** | **Identifier** |
| --- | --- | --- |
|  |  |  |

- 1. **Deposited data**

| **Name of repository** | **Identifier** | **Link** |
| --- | --- | --- |
|  |  |  |

- 1. **Software**

| **Software name** | **Manufacturer** | **Version** |
| --- | --- | --- |
| SPSS statistics | IBM | 28.0.1.0 x64 ENG |
| GraphPad Prism | GraphPad Software | 9.0.0.121 x64 ENG |
|  |  |  |
|  |  |  |

- 1. **Other (e.g. drugs, proteins, vectors etc.)**

|  |  |  |
| --- | --- | --- |
|  |  |  |

- 1. **Please provide the details of the corresponding methods author for the manuscript:**

| **Jesse Pustjens**  [**j.pustjens@erasmusmc.nl**](mailto:j.pustjens@erasmusmc.nl)  **1019 TX, Amsterdam**  **Baron G.A. Tindatstraat 108** |
| --- |
|  |

**2.0 Please confirm for randomised controlled trials all versions of the clinical protocol are included in the submission. These will be published online as supplementary information.**

|  |
| --- |
