# Supplementary material for: Differential prevalence and prognostic value of metabolic syndrome components among patients with MASLD
Source: JHEP Rep. 2024 Aug 22;6(12):101193. doi: 10.1016/j.jhepr.2024.101193 (PMC11617726; doi:10.1016/j.jhepr.2024.101193)
Supplement: Multimedia component 4 [file mmc4.pdf]

Research article

JHEP|Reports

Differential prevalence and prognostic value of metabolic syndrome components among patients with MASLD

Jesse Pustjens[1], Laurens A. van Kleef[1], Harry L.A. Janssen[1,2], Robert J. de Knegt[1], Willem P. Brouwer[1,*]

JHEP Reports **2024**. vol. 6 | 1–8

Check for updates

Background & Aims: Metabolic dysfunction-associated steatotic liver disease (MASLD) is becoming increasingly prevalent in the general population. This study aimed at describing the cardiometabolic burden of the MASLD population and to identify patients at the highest risk of all-cause mortality and liver fibrosis.

Methods: We analysed individuals with MASLD enrolled in the National Health and Nutrition Survey (NHANES) III study (N = 3,628) and in the NHANES 2017–2020 study (n = 2,618). MASLD was defined as hepatic steatosis (by ultrasonography or controlled attenuation parameter), together with cardiometabolic dysfunction. Primary endpoints were all-cause mortality and liver fibrosis (liver stiffness measurement ≥8 kPa). Regression models were adjusted for age, sex, race, marital status, education, and smoking, and results were stratified by age groups (20–40, 40–60, 60–80 years).

Results: Among the total MASLD population (median age = 48, [25th to 75th percentiles: 36–62] years, 44.8% males), 65% had three or more cardiometabolic disorders. The most frequent were obesity (89.1%), (pre-) diabetes (66.6%), and low-HDL (54.7%). During a median follow-up of 22.3 (25th to 75th percentiles: 16.9–24.2) years, 1,405 deaths occurred. Hypertension (adjusted hazard ratio [aHR] 1.42, 95% CI 1.26–1.61), (pre-)diabetes (aHR 1.28, 95% CI 1.09–1.49), and hypertriglyceridaemia (aHR 1.19, 95% CI 1.05–1.34) were the strongest predictors of all-cause mortality. Consistent results were obtained regarding the association between cardiometabolic disorders and fibrosis. Here, increased waist circumference (adjusted odds ratio [aOR] 3.45, 95% CI 1.44–8.25), (pre-)diabetes (aOR 1.90, 95% CI 1.44–2.25), and hypertension (aHR 1.84, 95% CI 1.40–2.43) showed the strongest associations.

Conclusions: MASLD patients vary greatly in their cardiometabolic burden and consequently, in their prognosis. Our results highlight MASLD as a disease spectrum rather than as a single disease entity, necessitating an individualised treatment approach.

© 2024 The Authors. Published by Elsevier B.V. on behalf of European Association for the Study of the Liver (EASL). This is an open access article under the CC BY license (http://creativecommons.org/licenses/by/4.0/).

Introduction

In recent years, metabolic-dysfunction associated steatotic liver disease (MASLD) has become one of the most prevalent causes of chronic liver disease, affecting more than 30% of the global population.[1] Although most MASLD cases do not progress to end-stage liver disease, its sheer prevalence has made it a prominent public health concern. Approximately 25% of patients with MASLD progress to metabolic-associated steatohepatitis (MASH), which in turn may progress to advanced liver disease, cirrhosis, hepatocellular carcinoma, and the need for liver transplantation.[2,3] Given the rising prevalence of metabolic risk factors in the coming years, MASH is expected to surpass alcoholic liver disease as the primary indication for liver transplantation.[2,4]

Unlike the metabolic dysfunction associated fatty liver disease (MAFLD) criteria, with the new MASLD criteria patients only need to meet one (instead of two) cardiometabolic dysfunction criteria in the presence of hepatic steatosis. Therefore, by the new nomenclature, an increased proportion of the general population is considered metabolically unhealthy.[5] While this high degree of inclusivity ensures adequate recognition of patients who potentially stand to benefit from cardiometabolic optimization, it also introduces a greater degree of heterogeneity within the MASLD population. The clinical and practical consequences of the steatotic liver disease (SLD) nomenclature change remain uncertain, as these do not exactly mirror the former non-alcoholic fatty liver disease (NAFLD) or the MAFLD population.

Considering the widespread and increasing prevalence of MASLD, it is important to differentiate between individuals who are at a relatively low risk of adverse outcomes from those facing the highest risks. Therefore, the purpose of this study is to provide a deeper understanding of the influence of cardiometabolic disorders on liver fibrosis and all-cause mortality within the MASLD population to aid healthcare professionals in understanding the heterogeneity of MASLD and in the recognition of at-risk patients.

* Corresponding author. Address: Department of Gastroenterology and Hepatology, Erasmus MC University Medical Centre, Dr. Molewaterplein 40, 3015 GD Rotterdam Na-6 (Mdl Secrt./Staf), Rotterdam, the Netherlands. Tel.: + 31628241861.
E-mail address: w.p.brouwe@erasmusmc.nl (W.P. Brouwer).
https://doi.org/10.1016/j.jhepr.2024.101193

Methods

Study populations

For this study, we used data from the National Health and Nutrition Examination Survey conducted from 1988 to 1994 (NHANES III), as well as the NHANES 2017-2020 database. Because of the distinct nature of the available data in each cohort, NHANES III was utilized to evaluate associations with all-cause mortality, whilst NHANES 2017–2020 was used for assessing associations with fibrosis. In short, NHANES is a program of studies designed to assess the health and nutritional status of the United States population. Periodically, a representative sample of individuals living in households across the United States are selected, of whom data is collected on various factors including, demographics, socioeconomic status, health-related questionnaires, physical examinations, laboratory tests, and imaging. Detailed information regarding the procedures and rationale has been described elsewhere.[6] We did not use the provided weights of the NHANES since this study was designed not to study prevalences, but associations between cardiometabolic disorders and mortality and fibrosis. Individuals with available data on both steatosis and alcohol use were considered eligible for inclusion. Exclusion criteria were missing data on age at baseline, lack of follow-up, and viral hepatitis. Participants with daily alcohol consumption ≥30 g in males or ≥20 g in females were excluded, in adherence to the MASLD definition. For the NHANES 2017-2020 specifically, participants with missing liver stiffness measurement (LSM) or controlled attenuation parameter (CAP) were excluded. Participants of the NHANES provided informed consent. This study was conducted in accordance with the principles of the Declaration of Helsinki.

MASLD

For NHANES III, hepatic steatosis was determined by abdominal ultrasonography (Toshiba Sonolayer SSA-90A, Tokyo, Japan). At inclusion, participants underwent ultrasound imaging, and the images were reassessed in 2009 and 2010 for the presence of hepatic steatosis. Ultrasonography is widely used as a screening tool for hepatic steatosis and has a good diagnostic performance with a sensitivity of 83.4%, specificity of 81.0%, and AUC of 0.82.[7] For the NHANES 2017-2020 CAP was used to define steatosis (CAP, ≥274 dB/m) as measured by FibroScan®.[8] CAP can be regarded superior to ultrasound in detecting hepatic steatosis with a sensitivity of 87%, a specificity of 91%, and a AUC of 0.96.[9] MASLD was identified if steatosis (irrespective of its degree) was present in coexistence with one or more cardiometabolic disorders, according to the SLD consensus statement.[10] These new criteria include 1) obesity (BMI ≥25 kg/m^2 or waist circumference [WC] >94 cm in males and >80 cm in females or an ethnicity-adjusted equivalent), 2) (pre-)diabetes (fasting serum glucose ≥5.6 mmol/L or 2-h post-load glucose levels ≥7.8 mmol/L, or HbA1c ≥5.7% or type 2 diabetes or treatment for type 2 diabetes, 3) hypertension (blood pressure ≥130/85 mmHg or specific antihypertensive drug treatment), 4) hypertriglyceridemia (plasma triglycerides ≥1.70 mmol/L or lipid lowering treatment or 5) low-HDL (high-density lipoprotein) (HDL ≤1.0 mmol/L in males and ≤1.3 mmol/L in females or lipid lowering treatment). For the purpose of the present study, we present results regarding obesity separately for BMI and WC, as BMI may have a paradoxical relationship with mortality.[11]

Covariates

Covariates were selected based on current scientific literature and included age, sex, race, marital status, education, and smoking status.[12] Data on covariates were systematically collected by the research assistants.

Alcohol

Data on alcohol consumption were retrieved using a food frequency questionnaire (FFQ) and nutritional interviews.[6] Participants were asked how many days of the year they drank alcoholic beverages and the average number of drinks consumed on drinking days. Data from the FFQ were compared to the interviews, and the highest amount was used for the analysis.

All-cause mortality

The mortality data of participants of the NHANES III cohort were obtained from the National Death Index provided by the National Center for Health Statistics, which contained complete data until the 31 December 2015.[13]

Liver fibrosis

In the NHANES 2017-2020 cohort, liver fibrosis was determined using FibroScan® (Echosens, France) model 502 V2 Touch, equipped with an M and XL probe. FibroScan® uses vibration-controlled transient elastography (VCTE) to derive liver stiffness. Measurements were considered valid if 10 consecutive measurements with (inter quartile range)/median of <30% were obtained. There is a good diagnostic performance of VCTE in diagnosing severe liver fibrosis compared with histological data obtained by biopsy.[14] In the present study, a value of ≥8 kPa was defined as significant liver fibrosis.[15]

Statistical analysis

Background characteristics were assessed using descriptive statistics and presented as means, 25th to 75th percentiles (P25–P75), standard deviations, frequencies, or percentages, according to the nature of the data. Cox proportional hazard models were used to estimate adjusted hazard ratios (aHR) and their respective 95% CI. Within the MASLD population, we examined the associations between specific cardiometabolic disorders and the number of cardiometabolic disorders with all-cause mortality, with the results divided into equally sized age groups of 20–40 years, 40–60 years, and 60–80 years. Third, multivariable linear and logistic regression modelling were used to test associations between cardiometabolic disorders and LSM as a continuous or dichotomized (LSM ≥8 kPa) variable. All models were adjusted for age, sex, race, marital status, education, and smoking status and included all cardiometabolic criteria in a dichotomized fashion. All analyses were performed SPSS v.28.0.1.0. p values <0.05 were considered statistically significant.

Results

Study cohort

The NHANES III cohort (1988–1994) consists of a total of 14,786 participants with available data on all-cause mortality. Participants with missing data on alcohol intake (n = 246), ultrasound data (n = 941), viral hepatitis (n = 428), or missing age data (n = 5) were excluded. Of the remaining 13,384 participants, 3,628 (27.1%) met the diagnostic criteria for MASLD, the baseline characteristics of which are shown in Table 1. The baseline characteristics of the MASLD population of the NHANES 2017–2020 cohort are presented in Table S1. In this cohort, 7,768 individuals were aged ≥18 years. Excluded were participants with missing data on alcohol intake (n = 400) or steatosis (n = 1). Of the remaining 7,367 participants, 2,618 (35.5%) met the MASLD criteria and were included in the final analysis.

Cardiometabolic risk factors

The frequencies of specific cardiometabolic disorders, as defined by the new SLD nomenclature, for the MASLD population in the NHANES III database are illustrated in Fig. 1. Of the MASLD population, 89.1% were obese and 66.6% were affected by (pre-)diabetes. The remaining cardiometabolic MASLD criteria, hypertension, hypertriglyceridemia, and low-HDL were observed in more than half of the MASLD population, with rates of 50.1%, 51.3%, and 54.7%, respectively. Approximately 65% of MASLD patients experience three or more cardiometabolic disorders. The prevalence and number of cardiometabolic disorders increased incrementally with increasing age. In adults aged 20–40 years, 83.1% were obese, 37.4% (pre)diabetic, 21.2% hypertensive, 39.4% had hypertriglyceridemia, and 58.5% had low-HDL levels, compared with 92.9%, 88.1%, 77.6%, 61.0%, and 51.8%, respectively, in adults aged 60–80 years. Similarly, among adults aged 20–40 years, 45.3% had three or more metabolic disorders, compared with 86.7% in adults aged 60–80 years.

Differential prognosis of cardiometabolic disorders

During the follow-up period (median 22.3 years [16.9–24.2]), 1,405 deaths were recorded in the MASLD population, resulting in a mortality rate of 19.5 per 1,000 person-years. In the multivariable analysis, hypertension, (pre-)diabetes, hypertriglyceridemia, and BMI were significantly associated with all-cause mortality (Table 2). Among the total MASLD population, the strongest predictor of all-cause mortality was hypertension (aHR 1.42, 95% CI 1.25–1.61), followed by (pre-)diabetes (aHR 1.28, 95% CI 1.09–1.49), and hypertriglyceridemia (aHR 1.19, 95% CI 1.05-1–34). When stratified by age categories (20–40, 40–60, and 60–80 years), the strength of the associations

Table 1. NHANES III participants characteristics stratified per age category.

	All age groups N = 3,628	Age 20–40 years n = 1,190	Age 40–60 years n = 1,295	Age 60–80 years n = 1,143
**Demographics**				
Age (years), median [P25-P75]	48.3 [34.9–61.7]	31.3 [26.4–36.2]	48.9 [43.7–54.1]	67.2 [63.4–71.0]
Male, n (%)	2,627 (44.8)	468 (39.3)	591 (45.6)	568 (49.7)
Race				
Non-Hispanic white	1,288 (35.5)	277 (23.3)	475 (36.7)	536 (46.9)
Non-Hispanic black	826 (22.8)	317 (26.6)	297 (22.9)	212 (18.5)
Mexican-American	1,369 (37.7)	545 (45.8)	461 (35.6)	363 (31.8)
Other	145 (4.0)	51 (4.3)	62 (4.8)	32 (2.8)
Years of education, median [IQR]	12.0 (10.0–14.0)	12 [10.0–14.0]	12 [9.5–14.5]	10 [7–13]
Current smoker, n (%)	767 (21.6)	316 (27.3)	284 (22.3)	167 (14.8)
Alcohol use (g/day), mean (SD)	3.1 (6.5)	3.6 (6.9)	3.1 (6.3)	2.5 (6.1)
**MASLD criteria, mean (SD)**	3.1 (1.3)	2.4 (1.2)	3.3 (1.2)	3.7 (1.1)
Obesity*	3,248 (89.5)	989 (83.1)	1,197 (92.4)	1,062 (92.9)
(Pre)Diabetes†	2,422 (66.8)	445 (37.4)	970 (74.9)	1,007 (88.1)
Hypertension, n (%)‡	1,818 (50.1)	252 (21.2)	679 (52.4)	887 (77.6)
Hypertriglyceridaemia§, n (%)	1,878 (51.8)	469 (39.4)	712 (55.0)	697 (61.0)
Low-HDL, n (%)**	1,997 (55.0)	696 (58.5)	709 (54.7)	592 (51.8)
**Biometrics**				
BMI (kg/m^2), median [IQR]				
Males	28.9 (25.8–32.1)	28.5 [25.15–31.9]	29.6 [26.3–32.9]	28.6 [25.9–31.4]
Females	30.0 (25.4–34.7)	29.2 [23.9–34.6]	30.9 [26.3–35.6]	29.9 [26.1–33.8]
Waist circumference (cm), median [IQR]				
Males	102.9 [94.8–111.1]	97.6 [88.4–106.9]	104.3 [96.4–112.2]	104.2 [97.0–11.5]
Females	98.8 [88.5–109.1]	93.3 [81.0–105.7]	100.4 [90.7–104.1]	101.7 [93.5–109.9]
**Biochemistry**				
ALT (U/L), median [IQR]	17.0 [10.5–23.5]	18.0 [9–27]]	18.0 [11.5–24.5]	15.0 [10.5–19.5]
AST (U/L), median [IQR]	20.0 [15.5–24.5]	20.0 [14.5–25.5]	20.0 [15.5–24.5]	20.0 [16–24]
HbA1c (%)	5.5 [5.1–6.0]	5.2 [4.9 -5.6]	5.6 [5.2–6.1]	5.8 [5.3–6.4]
Platelets	270.5 [224.3–316.8]	283 [237.4–328.7]	275.0 [229.8–320.3]	253.0 [208.6–297.5]

ALT, alanine aminotransferase; AST, aspartate aminotransferase; MASLD, metabolic-dysfunction steatotic liver disease; NHANES III, National Health and Nutrition Examination Survey (1988–1994); OR, odds ratio; P25-P75, 25th–75th percentile; WC, waist circumference. Data are presented as mean (SD), median [P25-P75], or n (%).
*BMI ≥25 kg/m^2 or WC >94 cm (males), >80 cm (females), or ethnically adjusted.
†Fasting serum glucose ≥5.6 mmol/L or 2-h post-load glucose levels ≥7.8 mmol/L or HbA1c. ≥5.7% OR type 2 diabetes or treatment for type 2 diabetes.
‡Blood pressure ≥130/85 mmHg or specific antihypertensive drug treatment.
§Plasma triglycerides ≥1.70 mmol/L or lipid lowering treatment.
**Plasma HDL-cholesterol ≤1.0 mmol/L (males) and ≤1.3 mmol/L (females) OR lipid lowering treatment.

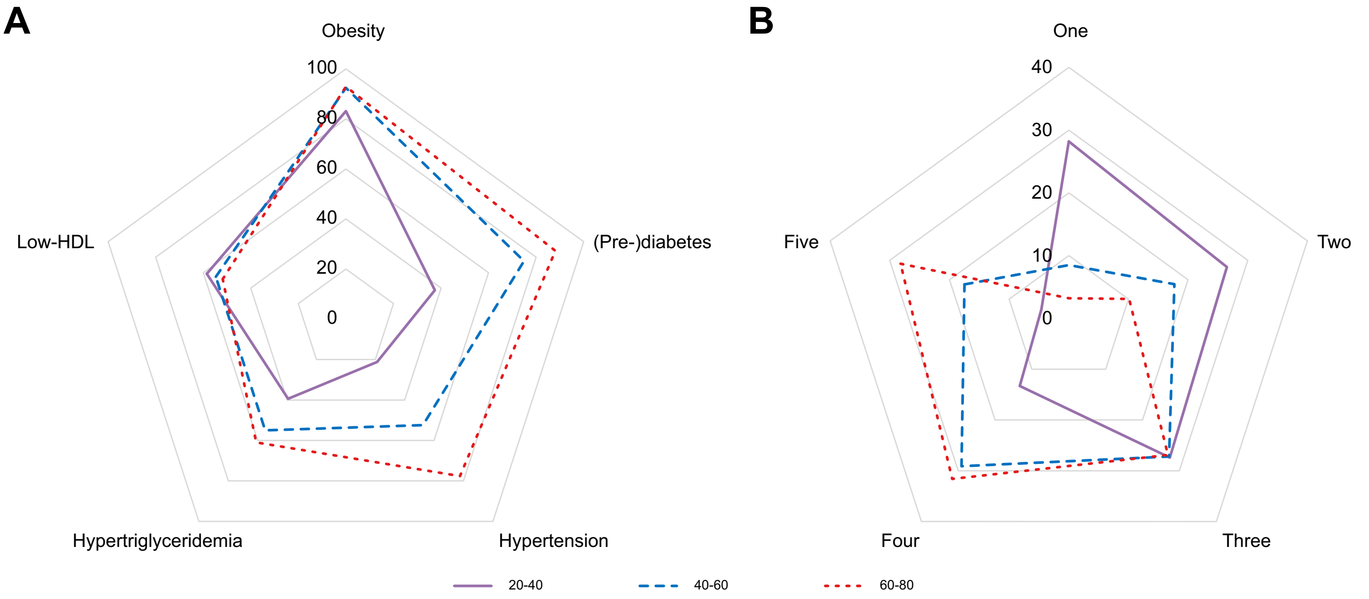

Fig. 1. The cardiometabolic burden among the MASLD population stratified per age category. (A) The prevalence of specific cardiometabolic disorders (%), (B) The prevalence of the cumulative number of cardiometabolic disorders (%). Data from the NHANES III dataset was used. MASLD, metabolic-dysfunction steatotic liver disease; NHANES III, National Health and Nutrition Examination Survey (1988–1994).

decreased with advancing age. The highest aHRs were obtained in adults aged 20-40 for hypertension (aHR 1.96, 95% CI 1.29–2.99), (pre-)diabetes (aHR 1.86, 95% CI 1.28–2.71) and hypertriglyceridaemia (aHR 1.65, 95% CI 1.12–2.44). In older adults (aged 60–80 years), associations with all-cause mortality and cardiometabolic disorders diminished and were no longer significant, except for hypertension, albeit with a decreased effect estimate (aHR 1.29, 95% CI 1.09–1.52). Increased BMI (≥25 kg/m^2) was inversely associated with all-cause mortality in adults aged 20–40 years (aHR 0.52, 95% CI 0.29–0.92) and 40-60 years (aHR 0.61, 95% CI 0.44–0.86).

Cumulative impact of metabolic risk factors

Higher mortality rates were observed for each additional cardiometabolic disorder (log-rank, p >0.001), as shown in Fig. 2. Consistent results were obtained using adjusted multivariable analysis (Table 3). Compared with the lowest possible cardiometabolic burden of one disorder in the MASLD population (as allowed by the MASLD definition), from three cardiometabolic disorders onwards, a positive association with all-cause mortality was observed. When all five cardiometabolic criteria were met, there was almost a double risk of all-cause mortality compared to having only one criterion met (aHR 1.92, 95% CI 1.47–2.51).

Impact of cardiometabolic disorders on liver stiffness

Different metabolic risk factors showed differential associations with LSM, both continuous and for values of ≥8 kPa (liver fibrosis). The highest aOR was observed for increased waist circumference (aOR 3.45, 95% CI 1.44–8.25), followed by (pre-)diabetes (aOR 1.90, 95% CI 1.44–2.52), hypertension (aOR 1.84, 95% CI 1.40–2.43), and low-HDL (aOR 1.51, 95% CI 1.20–1.91) (Table 4). A stepwise increase in aORs was observed with each additional cardiometabolic disorder, with five disorders resulting in the highest aOR (aOR 10.4, 95% CI 4.7–23.2) (Fig. 3).

Discussion

We used the newly adopted MASLD definition to characterise the cardiometabolic burden in the MASLD population. Our findings revealed that MASLD spans a wide disease spectrum that ranges from steatosis with a limited cardiometabolic burden to an extensive metabolic syndrome with five out of five criteria. Each of these cardiometabolic disorders has a differential association with all-cause mortality and liver fibrosis, and accumulation of multiple cardiometabolic disorders is associated with an increasingly poor prognosis.

Among the MASLD population, two-thirds had three or more cardiometabolic disorders, most commonly including obesity and (pre-)diabetes. Hypertension was identified as the most potent and consistent independent predictor of all-cause mortality across all age groups. Adults aged 20-40 years with hypertension exhibit a nearly two-fold increased risk of all-cause mortality compared with those without hypertension. Additionally, hypertriglyceridaemia shows a strong positive association in adults up to 40 years of age and (pre-)diabetes in adults up to 60 years of age. The varying prevalence and strength of these disorders as predictors of all-cause mortality are indicative of a complex interplay between cardiovascular disease and MASLD. Hepatic steatosis and the metabolic syndrome share important pathophysiological mechanisms, including insulin resistance, systemic inflammation, and oxidative stress.[16] It is therefore not surprising that patients with MASLD have been shown to have a decreased lifespan of approximately 4 years compared to those without, largely because of cardiovascular disease.[17–20] Interestingly, the relative role of hepatic steatosis in mortality remains a subject of debate. In a previous study, metabolically normal NAFLD individuals, demonstrated no difference in all-cause,

Table 2. All-cause mortality risk among the MASLD population in the for each cardiometabolic disorder, stratified per age category.

	All age groups N = 3,628			Age 20–40 years n = 1,190			Age 40–60 years n = 1,295			Age 60–80 year n = 1,143		
	aHR	95% CI	*p* value	aHR	95% CI	*p* value	aHR	95% CI	*p* value	aHR	95% CI	*p* value
**Metabolic disorder**												
BMI ≥25 kg/m^2	0.81	0.68–0.97	**0.018**	0.52	0.29–0.92	**0.025**	0.61	0.44–0.86	0.004	1.00	0.80–1.26	0.98
Increased waist circumference	1.10	0.89–1.36	0.36	1.31	0.74–2.31	0.35	1.60	1.05–2.45	**0.028**	0.91	0.69–1.20	0.49
(Pre-)diabetes	1.28	1.09–1.49	**0.002**	1.86	1.28–2.71	**0.001**	1.49	1.12–1.97	**0.005**	1.12	0.91–1.39	0.29
Hypertension	1.42	1.25–1.61	**<0.001**	1.96	1.29–2.99	**0.002**	1.57	1.26–1.96	**<0.001**	1.29	1.09–1.53	**0.003**
Hypertriglyceridemia	1.19	1.05–1.34	**0.005**	1.65	1.12–2.44	**0.011**	1.23	0.98–1.54	0.081	1.14	0.98–1.32	0.87
Low-HDL	1.06	0.95–1.19	0.31	0.99	0.66–1.46	0.94	1.09	0.87–1.37	0.46	1.07	0.92–1.23	0.38

aHR, adjusted hazard ratio; MASLD, metabolic-dysfunction steatotic liver disease; NHANES III, National Health and Nutrition Examination Survey (1988–1994). Analyses were performed in the NHANES III dataset. Results were obtained with Cox proportional hazards and are given as aHR with 95% CI for all-cause mortality as outcome. All cardiometabolic disorders were simultaneously added in the multivariate model and results were additionally adjusted for age, sex, race, marital status, years of education, smoking. *p* values <0.05 were considered statistically significant and are emboldened.

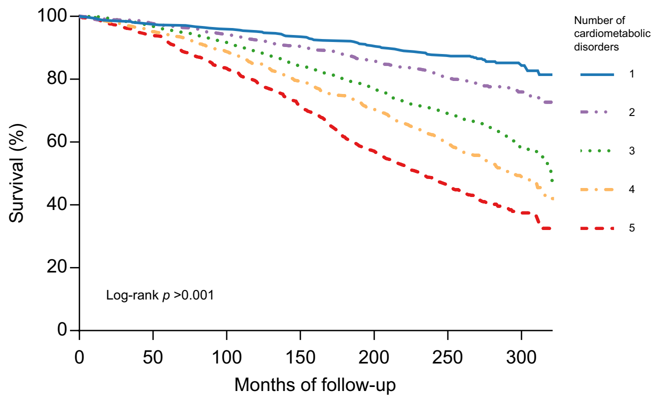

Fig. 2. Survival analysis of patients with MASLD stratified by the number of cardiometabolic disorders. Kaplan–Meier survival curves with a median follow-up of 22.9 [20.9–24.8] years of 3,628 MASLD patients, by cumulative number of cardiometabolic disorders (log-rank *p* >0.001). *p* values <0.05 were considered statistically significant. Data from the NHANES III study dataset were used. MASLD, Metabolic-dysfunction steatotic liver disease; NHANES III, National Health and Nutrition Examination Survey (1988–1994).

cardiovascular or liver-related mortality compared to metabolically normal individuals without hepatic steatosis.[17] Along the same line, others have proposed insulin resistance and central obesity as predictors of cardiovascular risk, rather than hepatic steatosis.[5] In an additional analyses performed in the present study, individuals with hepatic steatosis but without metabolic syndrome had a worse prognosis than those without hepatic steatosis but with metabolic syndrome. Potentially, hepatic steatosis presents a deep metabolic disturbance that, together with other cardiovascular disorders, drive mortality in the MASLD population. Strengthening this concept is the stepwise increase in all-cause mortality risk for each additional cardiometabolic disorder, as demonstrated in the present study.

Notably deviating from this trend is the seemingly protective effect of an increased BMI on all-cause mortality. However, this can be explained by the widely accepted phenomenon of ‘the obesity paradox,’ which recognizes BMI as an imperfect tool for defining obesity: Sub-obese BMIs do not only describe those with a healthy body composition but also those with sarcopenia and chronic disease, both of which are associated with poor prognoses.[11,21] WC has been proposed as a more accurate descriptor of central obesity, a notion supported by our results that demonstrate that an increased WC is associated with an increased risk of all-cause mortality in adults aged 40–60 years.

Another interesting finding is the age-dependent variation in our results. With advancing age, the strength of the association between cardiometabolic disorders and all-cause mortality attenuates. For example, hypertension has an aHR of 1.96 (95% CI 1.29–2.99) in those aged 20–40 years but lowers to 1.29 (95% CI 1.09–1.35) in the older population. Comparable results were seen in a study by van Kleef *et al.*, where hepatic steatosis was not associated with increased all-cause mortality in the older adults, whereas in younger cohorts, increased mortality has been described.[22,23] Several explanations for this trend are possible. Firstly, individuals presenting with cardiometabolic abnormalities at a younger age may experience a more profound metabolic disturbance than those who develop them later in life with a longer exposure duration, potentially reflecting

Table 3. All-cause mortality risk among the MASLD population for the number of cardiometabolic disorder, stratified per age category.

	All age groups N = 3,628			Age 20–40 years n = 1,190			Age 40–60 years n = 1,295			Age 60–80 years n = 1,143		
	aHR	95% CI	*p* value	aHR	95% CI	*p* value	aHR	95% CI	*p* value	aHR	95% CI	*p* value
Number of metabolic disorders												
1 criterion			Reference			Reference			Reference			Reference
2 criteria	1.21	0.91–1.62	0.20	0.69	0.37–1.28	0.24	1.07	0.63–1.82	0.81	1.78	1.12–2.85	**0.014**
3 criteria	1.46	1.12–1.91	**0.005**	1.79	1.09–2.95	**0.022**	1.36	0.83–2.24	0.23	1.72	1.11–2.66	**0.015**
4 criteria	1.55	1.19–2.01	**0.001**	1.80	1.01–3.22	**0.046**	1.77	1.09–2.88	**0.020**	1.71	1.11–2.63	**0.015**
5 criteria	1.92	1.47–2.51	**<0.001**	3.10	1.59–6.03	**0.001**	2.15	1.31–3.54	**0.003**	2.20	1.42–3.39	**<0.001**

aHR, adjusted hazard ratio; MASLD, metabolic-dysfunction steatotic liver disease; NHANES III, National Health and Nutrition Examination Survey (1988–1994). Analyses were performed in the NHANES III dataset. Results were obtained with Cox proportional hazards and are given as aHR with 95% CI for all-cause mortality as outcome. Results were adjusted for age, sex, race, marital status, years of education, smoking. *p* values <0.05 were considered statistically significant and are emboldened.

Table 4. Risk for at-risk liver fibrosis (LSM ≥8 kPa) and association with LSM (continuous) among the MASLD population for each specific metabolic disorder.

	LSM ≥8 kPa			LSM continuous		
	aOR	95% CI	*p* value	β	95% CI	*p* value
**Metabolic disorder**						
BMI ≥25 kg/m^2	1.83	0.94–3.56	0.075	0.58	-0.35–1.52	0.22
Increased waist circumference	3.45	1.44–8.25	**0.005**	1.19	0.18–2.19	**0.021**
(Pre-)diabetes	1.90	1.44–2.52	**<0.001**	0.64	0.16–1.12	**0.010**
Hypertension	1.84	1.40–2.43	**<0.001**	0.53	0.03–1.02	**0.037**
Hypertriglyceridaemia	0.99	0.77–1.26	0.91	0.39	-0.07–0.84	0.10
Low-HDL	1.51	1.20–1.91	**<0.001**	0.48	0.03–0.93	**0.037**

aOR, adjusted odds ratio; LSM, liver stiffness measurement; MASLD, metabolic-dysfunction steatotic liver disease; NHANES, National Health and Nutrition Examination Survey. Analyses were performed in the NHANES 2017-2020 dataset. Results were obtained with logistic and linear regression models and are given as aOR or β with 95% CI and liver stiffness as outcome. All cardiometabolic disorders were simultaneously added in the multivariate model and results were additionally adjusted for age, sex, race, marital status, years of education, smoking. *p* values <0.05 were considered statistically significant and are emboldened.

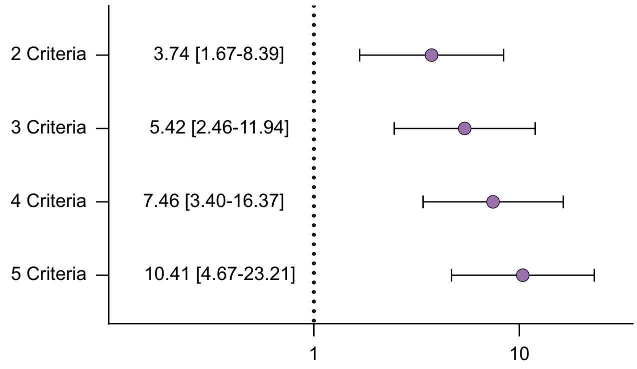

Fig. 3. Results obtained from logistic regression analyses for the risk fibrosis and the cumulative number of cardiometabolic disorders among the MASLD population. Plot of aOR on a logarithmic scale for the association of at-risk fibrosis (LSM ≥8 kPa) and the cumulative number of cardiometabolic disorders among the MASLD population, with one criterion as a reference. *p* values for two, three, four and five criteria were all <0.001. *p* values <0.05 were considered statistically significant. Data from the NHANES 2017-2020 study dataset were used. Model adjusted for age, sex, race, marital status, years of education, smoking. aOR, adjusted odds ratio; LSM liver stiffness measurement; NHANES, National Health and Nutrition Examination Survey.

a poorer clinical condition and, consequently, a poorer outcome. Secondly, comorbidities become increasingly common with advancing age. Older individuals often accumulate multiple health conditions, thereby reducing the relative impact of specific cardiometabolic disorders on all-cause mortality. Thirdly, the results may be affected by a survivor bias, whereby achieving a certain age may have selected those who demonstrate some level of resistance or adaptation to certain cardiometabolic disorders.

Besides cardiovascular disease, MASLD is a widely accepted risk factor for liver-related mortality and morbidity.[24] Similar to all-cause mortality, an increased cardiometabolic burden adversely affects the risk of fibrosis in a stepwise manner: for each additional cardiometabolic disorder added, relative risks increase. Similar results have been observed in the diabetic population, where an increase in the number of metabolic traits raises the risk of adverse liver outcomes.[25] In the current study, an increased WC emerged as the strongest predictor of fibrosis, but this warrants cautious interpretation. LSM as a proxy for fibrosis is a technique not free from limitations, and increased stiffness may reflect a higher degree of false positives in those with central obesity rather than true fibrosis.[26] Diabetes has been established as a strong risk factor for MASLD progression into MASH and concurrent fibrosis or cirrhosis, which is in agreement with our results.[27]

Our findings should be interpreted in light of the strengths and limitations of our study. A major strength of our study is that it is based on a large representative community-based cohort that includes both young and older participants. In addition, we had an extensive follow-up period, with a median length of 23 years, which enabled us to collect sufficient data on our primary endpoint; all-cause mortality. However, the long follow-up time also invites for time-dependent bias. Participants with cardiometabolic risk factors may have modified these factors during the follow-up period, which could have affected their outcome. Moreover, the metabolic burden of the described population, may not reflect the burden of today's population. However, there is no evidence to assume that

individual associations with metabolic syndrome components with mortality or fibrosis has changed. Furthermore, given the retrospective design of this study, the possibility of reverse causality cannot be excluded. Additionally, data on alcohol intake were collected using a FFQ and nutritional interviews, which carry the risk of a recall-bias. To address this, the highest self-reported intake (FFQ *vs.* interview) was used for the analyses. Nevertheless, without objective blood measures we cannot exclude the possibility that some patients with metabolic and alcohol related/associated liver disease (MetALD) were inadvertently included in our study. Furthermore, missing data on alcohol consumption may also be non-random, with abstinent individuals potentially leaving the alcohol intake question blank. The estimated effect size of this potential bias is limited, as only 11 participants in the NHANES III study and 76 in the NHANES 2017-2020 study who would otherwise have adhered to the MASLD definition were excluded. In a sensitivity analysis, we included participants with missing alcohol consumption data, although this did not impact the results. Lastly, a notable limitation is the lack of detailed data on fibrosis stages, which limits the ability to fully explore the clinical impact of our results.

Conclusion

The MASLD population is a heterogeneous group of patients exhibiting important differences in their cardiometabolic burden, which contributes to substantial differences in the risk of mortality and increased liver stiffness. Of the metabolic risk criteria, hypertension is the strongest predictor of all-cause mortality, especially in younger populations, followed by (pre-)diabetes and hypertriglyceridaemia. Moreover, the presence of multiple metabolic disorders exerts a detrimental impact on survival, with effects compounding as the metabolic syndrome expands. Therefore, it is important to identify cardiometabolic risk factors and recognise their distinct and increasing impact on the overall disease burden in clinical and research settings.

Affiliations

[1]*Department of Gastroenterology and Hepatology, Erasmus MC University Medical Centre, Rotterdam, The Netherlands;* [2]*Toronto Centre for Liver Disease, Toronto General Hospital, University Health Network, Toronto, Canada*

Abbreviations

aHR, adjusted hazard ratio; aOR, adjusted odds ratio; ALT, alanine aminotransferase; AST, aspartate aminotransferase; BMI, body mass index; CAP, controlled attenuation parameter; CI, confidence interval; FFQ, food frequency questionnaire; HDL, high-density lipoprotein; HbA1c, hemoglobin A1c; LSM, liver stiffness measurement; MAFLD, metabolic dysfunction associated fatty liver disease; MASH, metabolic dysfunction associated steatohepatitis; MASLD, metabolic dysfunction associated steatotic liver disease; MetALD, metabolic and alcohol-related liver disease; NAFLD, non-alcoholic fatty liver disease; NHANES, National Health and Nutrition Examination Survey; OR, odds ratio; P25-P75, 25th to 75th percentile; SLD, steatotic liver disease; VCTE, vibration-controlled transient elastography; WC, waist circumference.

Financial support

Financial support was provided by the Foundation for Liver and Gastrointestinal Research (Rotterdam, Netherlands). The funding source did not influence the study design, data collection, analysis, interpretation of the data, writing of the report, or the decision to submit for publication.

Conflicts of interest

JP: Full Bursary EASL congress 2024; LAvK: Travel Bursary EASL congress 2024; HLAJ: grants from Gilead Sciences, GlaxoSmithKline, Janssen, Roche, Vir Biotechnology Inc. and consultant for Aligos, Gilead Sciences, GlaxoSmithKline, Grifols, Roche, Vir Biotechnology Inc., Precision Biosciences; RJdK: Abbvie: Lectures, Research; Echosens and Consultancy, Lectures, Research; Gilead: Advisory Board, Lectures, Research; GSK: Research; Johnson & Johnson: Advisory Board, Research; Schallware: Lectures; WPB: Eli Lilly Speakers Fee; Novo Nordisk Advisory Board; Trials 89BIO, Boehringer Ingelheim, Novo Nordisk, and Inventiva Pharma.

Please refer to the accompanying ICMJE disclosure forms for further details.

Authors' contributions

Collection of data: JP, WPB, LAvK. Study design: JP, WPB. Data analysis: JP, WPB. Writing of the manuscript: JP. Critical review of the manuscript: WB, LAvK, HLAJ, and RJdK. Approval of the final version and approval of submission: all authors.

Data availability statement

Data are publicly available from the NHANES database (https://www.cdc.gov/nchs/nhanes/index.htm).

Acknowledgements

We gratefully acknowledge the contributions of the NHANES cohort participants, research assistants, and facilitating personnel.

Supplementary data

Supplementary data to this article can be found online at https://doi.org/10.1016/j.jhepr.2024.101193.

References

Author names in bold designate shared co-first authorship

[1] Le MH, Le DM, Baez TC, et al. Global incidence of non-alcoholic fatty liver disease: a systematic review and meta-analysis of 63 studies and 1,201,807 persons. J Hepatol 2023;79:287–295.
[2] Younossi ZM, Marchesini G, Pinto-Cortez H, Petta S. Epidemiology of nonalcoholic fatty liver disease and nonalcoholic steatohepatitis: implications for liver transplantation. Transplantation 2019;103:22–27.
[3] Younossi Z, Anstee QM, Marietti M, et al. Global burden of NAFLD and NASH: trends, predictions, risk factors and prevention. Nat Rev Gastroenterol Hepatol 2018;15:11–20.
[4] Devarbhavi H, Asrani SK, Arab JP, et al. Global burden of liver disease: 2023 update. J Hepatol 2023;79:516–537.
[5] Semmler G, Balcar L, Wernly S, et al. Insulin resistance and central obesity determine hepatic steatosis and explain cardiovascular risk in steatotic liver disease. Front Endocrinol (Lausanne) 2023;14:1244405.
[6] Plan and operation of the Third National Health and Nutrition Examination Survey. 1988-94. Series 1: programs and collection procedures. Vital Health Stat 1994;1:1–407.
[7] Lee CM, Yoon EL, Nakajima A, et al. A reappraisal of the diagnostic performance of B-mode ultrasonography for mild liver steatosis. Am J Gastroenterol 2023;118:840–847.
[8] Eddowes PJ, Sasso M, Allison M, et al. Accuracy of FibroScan controlled attenuation parameter and liver stiffness measurement in assessing steatosis and fibrosis in patients with nonalcoholic fatty liver disease. Gastroenterology 2019;156:1717–1730.
[9] Pu K, Wang Y, Bai S, et al. Diagnostic accuracy of controlled attenuation parameter (CAP) as a non-invasive test for steatosis in suspected non-alcoholic fatty liver disease: a systematic review and meta-analysis. BMC Gastroenterol 2019;19:51.
[10] Rinella ME, Lazarus JV, Ratziu V, et al. A multi-society Delphi consensus statement on new fatty liver disease nomenclature. J Hepatol 2023;20:1542–1556.

[11] Preston SH, Stokes A. Obesity paradox: conditioning on disease enhances biases in estimating the mortality risks of obesity. Epidemiology 2014;25:454–461.
[12] Kwak M, Kim HS, Jiang ZG, et al. MASLD/MetALD and mortality in individuals with any cardio-metabolic risk factor: a population based study with 26.7 years of follow-up. Hepatology 2024. https://doi.org/10.1097/HEP.0000000000000925.
[13] National Center for Health Statistics. Office of Analysis and Epidemiology, Public-use Linked Mortality File, 2015. Hyattsville, MD: National Center for Health Statistics; 2020 [cited 2022 Jul 10]. Available from: https://www.cdc.gov/nchs/data-linkage/mortality-public.htm. CDC. National Cancer for Health Statistics. National Health and Nutritional Examination Survey: analytic guidelines. http://www.cdc.gov/nchs/nhanes/nh3data.htm.
[14] Tsochatzis EA, Gurusamy KS, Ntaoula S, et al. Elastography for the diagnosis of severity of fibrosis in chronic liver disease: a meta-analysis of diagnostic accuracy. J Hepatol 2011;54:650–659.
[15] Roulot D, Costes JL, Buyck JF, et al. Transient elastography as a screening tool for liver fibrosis and cirrhosis in a community-based population aged over 45 years. Gut Jul 2011;60(7):977–984.
[16] Lonardo A, Nascimbeni F, Mantovani A, et al. Hypertension, diabetes, atherosclerosis and NASH: cause or consequence? J Hepatol 2018;68:335–352.
[17] Younossi ZM, Otgonsuren M, Venkatesan C, et al. In patients with non-alcoholic fatty liver disease, metabolically abnormal individuals are at a higher risk for mortality while metabolically normal individuals are not. Metabolism 2013;62:352–360.
[18] Golabi P, Otgonsuren M, de Avila L, et al. Components of metabolic syndrome increase the risk of mortality in nonalcoholic fatty liver disease (NAFLD). Medicine (Baltimore) 2018;97:e0214.
[19] Allen AM, Therneau TM, Larson JJ, et al. Nonalcoholic fatty liver disease incidence and impact on metabolic burden and death: a 20 year-community study. Hepatology 2018;67:1726–1736.
[20] Moon JH, Jeong S, Jang H, et al. Metabolic dysfunction-associated steatotic liver disease increases the risk of incident cardiovascular disease: a nationwide cohort study. EClinicalMedicine 2023;65:102292.
[21] Curcic IB, Berkovic MC, Kuna L, et al. Obesity paradox in chronic liver diseases: product of bias or a real thing? J Clin Transl Hepatol 2019;7:275–279.
[22] van Kleef LA, Sonneveld MJ, Kavousi M, et al. Fatty liver disease is not associated with increased mortality in the elderly: a prospective cohort study. Hepatology 2023;77:585–593.
[23] Simon TG, Roelstraete B, Khalili H, et al. Mortality in biopsy-confirmed nonalcoholic fatty liver disease: results from a nationwide cohort. Gut 2021;70:1375–1382.
[24] Younossi ZM, Koenig AB, Abdelatif D, et al. Global epidemiology of nonalcoholic fatty liver disease-Meta-analytic assessment of prevalence, incidence, and outcomes. Hepatology 2016;64:73–84.
[25] Shang Y, Grip ET, Modica A, et al. Metabolic syndrome traits increase the risk of major adverse liver outcomes in Type 2 diabetes. Diabetes Care 2024;47:978–985.
[26] Wong GL, Wong VW, Chim AM, et al. Factors associated with unreliable liver stiffness measurement and its failure with transient elastography in the Chinese population. J Gastroenterol Hepatol 2011;26:300–305.
[27] Targher G, Corey KE, Byrne CD, et al. The complex link between NAFLD and type 2 diabetes mellitus - mechanisms and treatments. Nat Rev Gastroenterol Hepatol 2021;18:599–612.

Keywords: Steatotic liver disease; Mortality; Fibrosis; Metabolic dysfunction associated fatty liver disease; Metabolic-dysfunction associated steatotic liver disease; Non-alcoholic fatty liver disease.
Received 22 March 2024; received in revised form 30 July 2024; accepted 5 August 2024; Available online 22 August 2024

Journal of Hepatology, Volume *6*

Supplemental information

Differential prevalence and prognostic value of metabolic syndrome components among patients with MASLD

Jesse Pustjens, Laurens A. van Kleef, Harry L.A. Janssen, Robert J. de Knegt, and Willem P. Brouwer

Differential prevalence and prognostic value of metabolic syndrome components among patients with MASLD

Jesse Pustjens, Laurens A. van Kleef, Harry L.A. Janssen, Robert J. de Knegt, Willem P. Brouwer

Table of content

Table S1……………………………………………………………………………………2

Table S1: NHANES 2017-2020 participants characteristics among the MASLD population

	N = 2618
**Demographics**	
Age (years), median [QR]	52.9 [40.9 – 64.9]
Male, N (%)	1,424 (54.4)
Race	
- White	954 (36.4)
- Black	534 (20.4)
- Hispanic	714 (27.3)
- Asian	284 (10.8)
- Other	132 (5.0)
Years of education, median	
- <9th grade	210 (8.2)
- 9th-11th grade	260 (10.1)
- High school	636 (24.8)
- College graduate	1,458 (55.9)
Currently smoking, N (%)	361 (13.8)
Alcohol use (g/day), mean (SD)	2.89 (5.6)
**MASLD criteria, mean (SD)**	3.28 (1.2)
Obesity [a]	2565 (98.0)
(pre)Diabetes [b]	1730 (66.1)
Hypertension, N (%) [c]	1664 (63.6)
Hypertriglyceridemia , N (%) [d]	1565 (59.8)
Low-HDL, N (%) [e]	1062 (40.6)
**Biometrics**	
BMI (Kg/M^2), median [IQR]	
- Males	31.6 [28.3 – 25.6]
- Females	33.4 [29.0 – 39.5]
Waist circumference (cm), median [IQR]	
- Males	
- Females	109.6 [101.0 – 119.9]
**Biochemistry**	
ALT (U/L), median [IQR]	21 [15 – 31]
AST (U/L), median [IQR]	20 [16 – 25]
HbA1c (%), median [IQR]	6 [6-6]
Platelets	243 [205 – 285]
**FibroScan®**	
Liver Stiffness Measurements (kPa)	5.6 [4.5 – 7.0]
Controlled Attenuation Parameter (dB/m)	316 [294 – 347]

[a] BMI ≥ 25 kg/m^2 or WC > 94 cm Waist circumference >94 cm (males), > 80 cm (females), or ethnically adjusted
[b] Fasting serum glucose ≥ 5.6 mmol/L or 2-hour post-load glucose levels ≥ 7.8 mmol/L or HbA1c ≥ 5.7% OR type 2 diabetes or treatment for type 2 diabetes
[c] Blood pressure ≥ 130/85 mmHg or specific antihypertensive drug treatment
[d] Plasma triglycerides ≥ 1.70 mmol/L or lipid lowering treatment
[e] Plasma HDL-cholesterol ≤ 1.0 mmol/L (males) and ≤ 1.3 mmol/L (females) OR lipid lowering treatment

Note: Data are presented as mean (SD), median [P25-P75], or n and percentage.
Abbreviations: NHANES, National Health and Nutrition Examination Survey; MASLD, metabolic-dysfunction steatotic liver disease; ALT, alanine aminotransferase; AST, aspartate aminotransferase; P25-P75, 25th–75th percentile;
